# Transient but Significant Visual Field Defects after Robot-Assisted Laparoscopic Radical Prostatectomy in Deep Trendelenburg Position

**DOI:** 10.1371/journal.pone.0123361

**Published:** 2015-04-23

**Authors:** Yukako Taketani, Chihiro Mayama, Noriyuki Suzuki, Akiko Wada, Tatsuhiro Oka, Kazuya Inamochi, Yohei Nomoto

**Affiliations:** 1 Department of Ophthalmology, The University of Tokyo Graduate School of Medicine, Tokyo, Japan; 2 Department of Ophthalmology, Asahi General Hospital, Chiba, Japan; 3 Department of Urology, Asahi General Hospital, Chiba, Japan; 4 Department of Anesthesiology, Asahi General Hospital, Chiba, Japan; 5 Department of Ophthalmology, Tokyo Metropolitan Police Hospital, Tokyo, Japan; University of Melbourne, AUSTRALIA

## Abstract

**Background:**

Robot-assisted laparoscopic radical prostatectomy (RALP) is a minimally invasive surgical procedure for prostate cancer. During RALP, the patient must be in a steep Trendelenburg (head-down) position, which leads to a significant increase in intraocular pressure (IOP). The association of RALP with visual field sensitivity, however, has not been prospectively studied. The purpose of this study was to evaluate prospectively the visual field, retinal nerve fiber layer (RNFL) thickness, and optic disc morphology in 50 normal eyes of 25 male patients that underwent RALP.

**Methods:**

The subjects were 25 males among 33 consecutive patients who underwent uneventful RALP under general anesthesia in our hospital. Visual field tests using the Humphrey visual field analyzer 30-2 SITA-standard program were performed before, 7 days after, and 1-3 months after RALP. IOP was measured before, during, and after RALP; and ophthalmologic examinations, including slit-lamp, fundus examination, and optical coherence tomography (OCT), were scheduled before and 7 days after surgery.

**Results:**

IOP was significantly increased during RALP up to 29.4 mmHg (P<0.01). Postoperative local visual field defects were detected in 7 eyes of 7 subjects dominantly in the lower hemifield without abnormal findings in the optic nerve head or retina, and the visual field recovered to normal within 3 months after surgery. General factors associated with RALP, IOP, RNFL thickness, or optic disc parameters did not differ significantly between eyes with and without postoperative visual field defects, and parameters of OCT measurements were not altered after surgery.

**Conclusion:**

Transient but significant unilateral visual field defects were found in 28% of the subjects examined. The probable cause are the increased IOP and altered perfusion during surgery and ophthalmologic examinations are therefore suggested before and after RALP.

## Introduction

Robot-assisted laparoscopic radical prostatectomy (RALP) is commonly performed in many countries, with more than 114,000 cases performed worldwide in 2013. Prostate cancer is one of the most common cancers in men, and RALP, as a relatively new surgical treatment, has several advantages, including less blood loss and pain. [1] The steep Trendelenburg position (head-down position up to 45°), which may be maintained for hours under general anesthesia during RALP, however, is associated with putative physiologic risk. Postoperative visual field loss has been recognized as a serious complication, and steep Trendelenburg position may cause it by significant intraocular pressure (IOP) elevation during the surgery. [2] Increased IOP, [3,4] arterial pressure, and central venous pressure [4,5] are observed during RALP, and high IOP and impaired perfusion may harm the optic disc, optic nerve, or retina. Glaucoma and ischemic optic neuropathy (ION) are possible complications of raised IOP during operation as suggested after refractive surgeries [6,7] and could result in serious visual loss.

The influence of the RALP procedure on visual function has not been studied in detail. There are two reported cases of posterior ION (PION) with bilateral permanent visual field defects or loss of light perception after minimally invasive prostatectomy, one of them performed using RALP. [8] On the other hand, a prospective study in which IOP, retinal nerve fiber layer (RNFL) thickness, and visual acuity were examined before and after RALP, reported no significant change in RNFL thickness or visual acuity, despite a significantly increased IOP during the procedure. [9] Visual field sensitivity after RALP, however, has not been prospectively studied.

The purpose of the present study was to evaluate the effect of RALP on the visual field in normal eyes. Visual field, RNFL thickness, and optic disc morphology were evaluated before and after RALP using static automated perimetry and optical coherence tomography (OCT). IOP was also measured during the procedure.

## Materials and Methods

This single-center, prospective, non-randomized study was conducted according to the tenets of the Declaration of Helsinki and received approval by the Institutional Review Broad of Asahi General Hospital. Written informed consent was obtained from each subject after explanation of the study protocol. A total of 33 consecutive male patients who underwent RALP under general anesthesia in Asahi General Hospital from February to December in 2013 were initially included, but 8 were excluded for reasons described below, and thus 25 patients were the subjects of the study.

The paired t-test was applied to examine differences in the values before and after surgery, and an unpaired t-test was applied otherwise. Repeated-measures analysis of variance and a paired t-test were applied to examine the changes in IOP. A P value <0.05 was considered statistically significant after Bonferroni’s correction for multiple comparisons. All statistical analyses were carried out using SPSS software (Version 19.0, IBM Software JAPAN, Tokyo).

### Indication for prostatectomy

RALP was performed by a single surgeon and the indication criteria for the surgery were the same as for conventional prostatectomy (cases with prostate specific antigen [PSA] <10–30 ng/ml, Gleason Score <9, and clinical stage T1 to T2). Patients with a history of cerebral infarction, cerebral hemorrhage, and abdominal surgery (other than appendicitis, inguinal hernia repair, or laparoscopic cholecystectomy) were excluded from the study.

### General anesthesia procedures and status monitoring

The anesthesia protocol was standardized for all subjects (ASA physical status I-II). No pre-medications were used. The patients were anesthetized with propofol (target controlled infusion, 2–4 μg/ml) or desflurane (3–5%), in combination with continuous infusion of remifentanyl (0.1–1.0 μg/kg/min) to maintain blood pressure, heart rate, and bispectral index. Rocuronium was continuously (20 mg/h) or repeatedly administrated for muscle relaxation. The lung was mechanically ventilated to maintain end-tidal carbon dioxide (ET CO_2_) concentration and airway pressure. The head-down position was set to 25° or 30°, according to the physical constitution of the patient and surgeon preference. To avoid facial compression, face masks specifically intended for use in the prone position were used. Blood pressure was measured via an arterial cannula inserted into the right radial artery or a cuff on the left brachium.

Blood pressure, heart rate, peak airway pressure, oxygen saturation, ET CO_2_, and bispectral index were continuously monitored, and IOP was measured before, after, and during surgery, as described later. Blood samples were collected before RALP and the day after RALP, and amount of blood loss was estimated based on body weight, hemoglobin level, and the amounts of infused fluid and colloid solution because direct measurement of blood loss during RALP is theoretically difficult due to interfusion of perfused fluid and urine in the abdominal cavity.

### Ophthalmologic examinations

All patients enrolled in the study underwent the following ocular examinations 0–2 months before RALP: visual acuity (VA) test, IOP measurement using the Goldmann applanation tonometer, slit-lamp biomicroscopy, indirect funduscopy under pupil dilation with topical tropicamide and phenylephrine (Midorin-P, Santen Pharmaceutical Co., Ltd., Osaka, Japan), and static automated visual field measurement using the Humphrey visual field analyzer (HFA) with the 30–2 SITA-standard program (Carl-Zeiss Meditec Inc., Dublin, CA). Circumpapillary RNFL thickness was also analyzed by OCT (Cirrus HD-OCT model 4000, Carl Zeiss Meditec Inc.) and fundus photographs (TRC, Topcon Inc., Tokyo, Japan) were obtained under pupil dilation. Exclusion criteria included best corrected visual acuity <20/20, ocular hypertension (IOP >21 mmHg), abnormal visual field test results (described later), presence or history of any eye diseases that might affect IOP or visual field testing (including corneal disease, cataract, glaucoma, diabetic retinopathy, and retinal vein occlusion), or history of intraocular surgery (except uncomplicated cataract surgery) or refractive surgery. Patients with a history of intracranial and/or central nervous system disease, such as cerebral infarction or hemorrhage, were also excluded.

An abnormal visual field was defined as a cluster of three or more points in a pattern deviation plot within a single hemifield (superior or inferior, except most peripheral points) with P values <5%, one of which must have a P value <1% according to Anderson and Patella’s criteria. [10] When a significant defect was observed only in the pattern deviation plot (not in the total deviation plot) and the Glaucoma Hemifield Test result was normal, the results of the total deviation were substituted for the pattern deviation in consideration of the limited experience of the subjects in visual field tests.

All the examinations mentioned above were conducted 0–2 months before RALP and repeated again approximately 7 days (range, 5–9 days) after the procedure. Only patients whose both eyes fulfilled the criteria were included in the study.

### IOP measurement

IOP of both eyes was measured using the Tono-pen XL (Medtronic Ophthalmic Incorporation, Jacksonville, FL) 8 times in each subject throughout the study period under topical anesthesia of a singular drop of 0.4% oxybuprocaine if conscious (Table 1). IOP was measured 1 day before RALP in the supine position (P1), during RALP in the horizontal supine position 5 min after intubation under systemic anesthesia (P2), at four discrete time points (10 min, and 1, 2.5, and 4 hours; P3–P6) after the head was lowered 25–30°, 10 min after the body was returned to the horizontal supine position (P7), and the next day in the supine position (P8).

**Table 1:** Time points of intraocular pressure measurements and condition of the subjects.

Time points	Condition
P1: Day before RALP	Supine position, conscious
P2: 5 min after systemic anesthesia induction	Horizontal supine position, anesthetic
P3: 10 min after maintaining head-down positon	Head-down supine position, anesthetic, with insufflation of the abdomen with CO_2_.
P4: 1 h after maintaining head-down positon	Head-down supine position, anesthetic, with insufflation of the abdomen with CO_2_
P5: 2.5 h after maintaining head-down positon	Head-down supine position, anesthetic, with insufflation of the abdomen with CO_2_
P6: 4 h after maintaining head-down positon	Head-down supine position, anesthetic, with insufflation of the abdomen with CO_2_
P7: 10 min after release of head-down position	Horizontal supine position, anesthetic
P8: Day after RALP	Supine position, conscious

RALP: Robot-assisted laparoscopic radical prostatectomy.

All IOP measurements were obtained by the same examiner for each subject. The tonometer was calibrated according to the manufacturer’s guidelines before the measurements and the mean value of four reliable measurements with standard error ≤5% was adopted at each time point. In all eyes, the difference in IOP measured using the Tono-pen XL and Goldmann applanation tonometer obtained in the sitting position before RALP was 2 mmHg or less.

Ocular perfusion pressure was calculated as mean arterial pressure minus IOP. Mean OPP value or time-integrated value during the surgery was used in the analyses.

### Visual field examination

Each subject underwent static automated perimetry twice using the HFA 30–2 SITA-standard program at 0–2 months before RALP. Due to the learning effect, we adopted the results of the second test. If the test result was unreliable (false-positive, false-negative, or fixation loss >30%), it was discarded and a re-test was administered on the same day. Visual field test was repeated in the same manner at approximately 7 days after RALP, and the test result was confirmed with another test within 1 week if a reliable postoperative visual field result was not obtained. Eyes with a significant postoperative visual field defect underwent another visual field test 1–3 months after RALP, and further tests were scheduled every 3 months after that until the visual field returned to normal.

Mean deviation (MD), pattern standard deviation (PSD), foveal threshold, and the mean value of total deviation (TD) in all test points in the superior or inferior hemifield were calculated and analyzed.

### RNFL measurement

RNFL thickness was measured using the Cirrus HD-OCT 0–2 months before and 7–10 days after RALP. Images with signal strength ≤5 were discarded and re-examined. Among the retinal parameters, average RNFL thickness, superior RNFL thickness, and inferior RNFL thickness were included in the analyses. Disc area, rim area, average C/D ratio, and cup volume were also included as parameters of optic nerve head morphology.

## Results

Of 33 patients that underwent RALP during the study period, 8 were excluded after the ophthalmologic examinations due to unilateral and bilateral abnormal visual fields in 4 and 1, respectively, with large optic disc cuppings suggesting pre-existing glaucoma, blood vein occlusion, or other pathologic changes. Visual acuity was <20/20 and/or reliable visual field or OCT results were not available for the other 3 patients, due to significant cataract. There were 3 other eyes with clusters of abnormal points in the pattern deviation plots, but these were judged as normal visual fields because their total deviation plots and Glaucoma Hemifield Test results were normal and the initial postoperative pattern deviation plots had been normal.

Fifty eyes of 25 subjects aged 52–74 years were finally included in the study and their characteristics are shown in Table 2. All subjects underwent uneventful RALP. The initial postoperative visual field tests were performed 7.0±2.2 (ranged 5–14) days after surgery when none of the subjects had ocular symptoms or abnormal findings in the ophthalmologic examinations including corneal erosion or edema or hemorrhage in the optic disc or retina. Among them, three eyes of two subjects showed unreliable visual field test results, which were discarded. The results obtained at 14 days after surgery were adopted in those two subjects. No eyes showed 2 or more lines of deterioration in visual acuity.

**Table 2:** Demographic data and parameters of the visual field, retinal nerve fiber layer thickness, and optic disc morphology of the subjects.

	VF defect (-)	VF defect (+)	Total
N (eye/person)	43/18	7/7	50/25
Age (year)	63.8±5.7	64.4±7.1	64.0±6.0
Gender(M/F)	18 Male	7 Male	25 Male
BMI	23.5±2.1	25.2±2.4	24.0±2.3
VF interval (day)	6.8±2.2	7.0±3.4	7.0±2.2
Operation time (hour)	5.4±1.0	5.2±0.9	5.3±1.0
Blood loss (ml)	241±172	334±227	267±188
Infusion volume (ml)	1671±532	1891±570	1733 ±534
Mean blood pressure (mmHg)*	83.5±6.7	76.9±8.9	81.5±7.9
Integral OPP (mmHg▪min)**	14287±5394	11250±3436	13406±4963

VF defect: Visual field defect after robot-assisted laparoscopic radical prostatectomy

Gender(M/F): Gender(Male/Female)

BMI: Body mass index.

VF interval: Time interval between the surgery and the initial visual field test

Operation time: Duration of robot-assisted laparoscopic radical prostatectomy

OPP: Ocular perfusion pressure; OPP = mean arterial pressure—intraocular pressure

* Mean blood pressure is an average value of the mean arterial pressure during the surgery.

**OPP is calculated as mean values of the both eyes in the 18 subjects without VF defect and mean values of the unilateral 7 eyes with VF defect in the other 7 subjects and integrated during the operation time.

**Fig 1:**
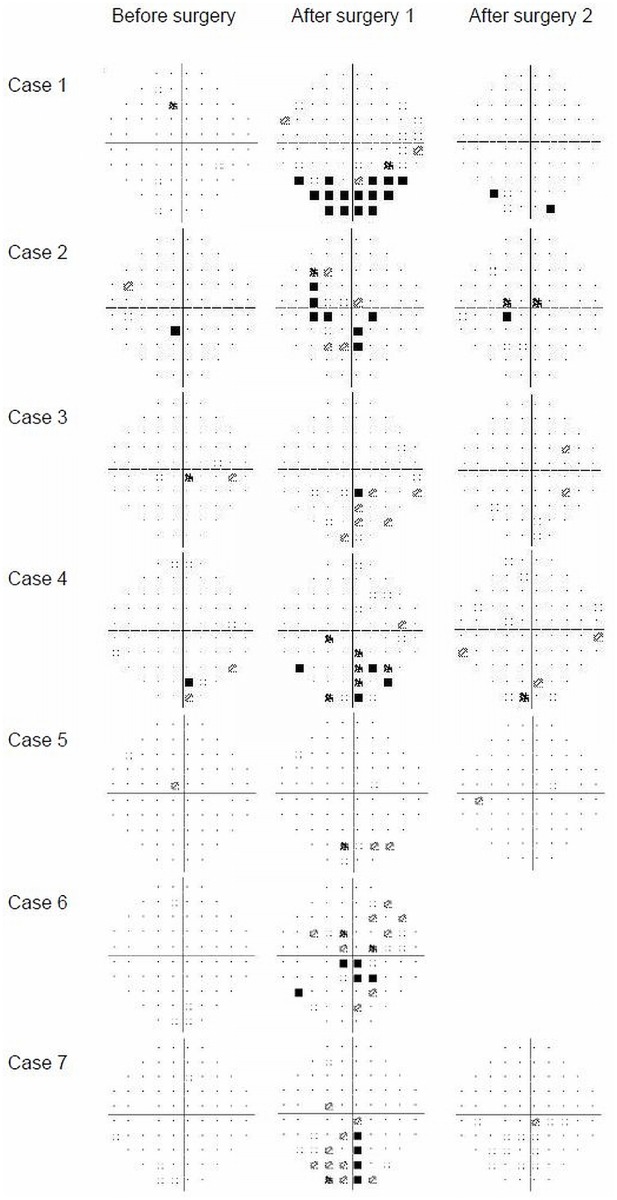
Visual fields of seven eyes of seven cases with unilateral postoperative visual field defects. Before surgery: 0–2 months before surgery. After surgery 1: mean of 7.0 days after surgery. After surgery 2: mean of 77 days after surgery.

Seven eyes of 7 subjects showed significant visual field defects in the initial postoperative examinations. All 7 eyes showed clusters of three or more abnormal points in the pattern deviation plot and four of them also showed Glaucoma Hemifield Test results of “Outside normal limits”. Five patients had a visual field defect in the lower hemifield (cases 1,3,4,5, and 7; Fig 1) and the other two patients had both upper and lower hemifield defects (cases 2 and 6). Visual field tests performed a mean of 77 days after the surgery, except in one subject who refused to take the test, indicated that the visual field had returned to normal.

**Fig 2:**
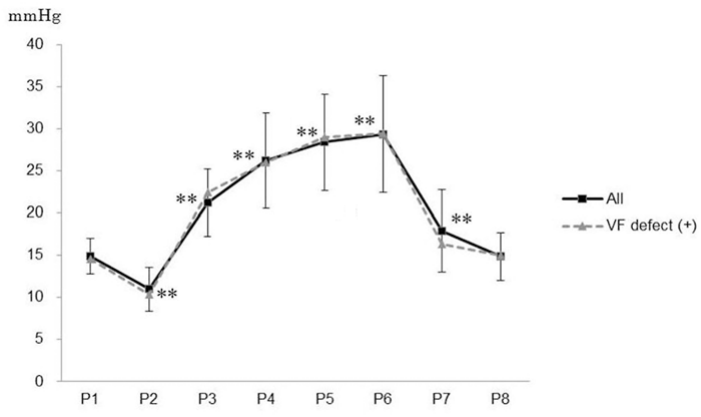
Intraocular pressure of at each time point. P1: Day before RALP (conscious).P2: 5 min after systemic anesthesia induction.P3: 10 min after maintaining head-down position.P4: 1 hour after maintaining head-down position.P5: 2.5 hours after maintaining head-down position.P6: 4 hours after maintaining head-down position.P7: 10 min after release of head-down position.P8: Day after RALP (conscious).All: 50 eyes of all 25 subjects.VF defect +: 7 eyes with postoperative visual field defects.Error bars represent standard deviations of IOP of all subjects’ eyes.**: P<0.01, compared with the value at P1, after Bonferroni’s correction.

Mean IOP did not differ significantly between the 7 eyes with and the 43 eyes without postoperative visual field defects at any of the 8 time points (Fig 2). Mean IOP of all 50 eyes was 14.9±2.1 mmHg preoperatively, and it significantly decreased to 11.0±2.7 mmHg at P2, 5 min after the induction of systemic anesthesia in the horizontal supine position (P<0.01). IOP significantly increased to 21.2±4.0 mmHg 10 min after the patient assumed the Trendelenburg position (P3, P<0.01) and further increased as the operation time advanced (P4–P6, P<0.01) up to 29.4±7.0 mmHg (P6). IOP decreased to 17.9±4.9 mmHg 10 min after the patient was returned to the horizontal position (P7), remaining significantly higher than the preoperative measurement values (P<0.01), P1, and returned to the preoperative level the next day (14.8±2.8 mmHg, P8).

In anesthesia, desflurane was chosen in 23 cases and propofol was used in 2 cases, one with postoperative visual field defect and one without it. Mean IOP during the surgery was not significantly different between those two groups: 21.3 and 20.1 mmHg in the right eyes and 21.9 and 19.5 mmHg in the left eyes, respectively.

The parameters of surgery, visual field test, and OCT did not differ significantly between groups with and without visual field defects or before and after RALP after Bonferroni’s correction for multiple comparisons (Tables 2 and 3). The only exception was PSD in all subjects, which was significantly increased after RALP (P = 0.034, after Bonferroni’s correction). Morphologic parameters in the retina and optic disc were also studied using OCT, and neither RNFL thickness nor any of disc parameters differed significantly between those with and without postoperative visual field defects or before and after RALP (Table 2).

**Table 3:** Demographic data and parameters of the visual field, retinal nerve fiber layer thickness, and optic disc morphology of the subjects.

		VF defect (-)	VF defect (+)	Total
Before RALP	MD (dB)	0.17±1.20	-0.40±0.95	0.09±1.17
	PSD (dB)	1.88±0.65	1.91±0.39	1.89±0.62
	Superior mean TD (dB)	0.15±1.7	-0.03±1.4	0.13±1.7
	Inferior mean TD (dB)	0.07±1.2	-0.76±1.0	-0.04±1.2
	Foveal threshold (dB)	36.6±2.1	37.3±2.1	36.7±2.1
	Average RNFLT (μm)	88.6±8.9	94.8±7.5	89.4±8.9
	Superior RNFLT (μm)	104.3±20.4	124.3±7.4	106.8±20.3
	Inferior RNFLT (μm)	114.4±15.6	122.2±13.9	115.3±15.5
	Disc area (mm^2^)	2.03±0.36	2.15±0.36	2.04±0.36
	Rim area (mm^2^)	1.31±0.23	1.33±0.15	1.31±0.22
	Vertical C/D ratio	0.51±0.15	0.52±0.10	0.51±0.14
	Cup volume (mm^3^)	0.21±0.18	0.21±0.09	0.21±0.17
After RALP	MD (dB)	0.28±1.38	-1.56±1.64	0.03±1.54
	PSD (dB)	2.25±1.08	3.48±1.83	2.42±1.26*
	Superior mean TD (dB)	0.41±2.0	-0.36±1.5	0.30±1.9
	Inferior mean TD (dB)	-0.05±1.7	-2.8±2.9	-0.43±2.1
	Foveal threshold (dB)	36.4±2.4	37.4±1.9	36.5±2.3
	Average RNFLT (μm)	91.0±9.1	95.4±7.1	91.7±8.9
	Superior RNFLT (μm)	109.0±15.6	118.0±12.2	110.4±15.3
	Inferior RNFLT (μm)	118.4±19.9	127.9±12.3	119.9±19.1
	Disc area (mm^2^)	2.02±0.38	2.14±0.36	2.04±0.37
	Rim area (mm^2^)	1.29±0.27	1.30±0.14	1.29±0.25
	Vertical C/D ratio	0.51±0.17	0.53±0.11	0.51±0.16
	Cup volume (mm^3^)	0.23±0.21	0.24±0.12	0.23±0.19

VF defect: Visual field defect after robot-assisted laparoscopic radical prostatectomy

MD: Mean deviation PSD: Pattern standard deviation TD: Total deviation

RNFLT: Retinal nerve fiber layer thickness

*: P<0.05 after Bonferroni's correction between before and after RALP.

## Discussion

Previous studies discussed the risk of substantial visual loss after systemic surgical procedures, including spinal surgeries, [11,12,13] coronary artery bypass, [14,15] and prostate surgery. [8,16] Visual loss may occur with or without evident findings in the optic disc, such as edema or reddening, and the cause of sudden visual loss is believed to be non-arteritic ischemic optic neuropathy (NAION). Visual loss due to NAION after systemic surgery results in severe and sometimes total visual field loss. It often occurs bilaterally, and visual function improves little in most cases lacking effective treatment options.

ION is classified into two types: anterior ION and posterior ION (PION). PION may occur after RALP [8] and involves the posterior part of the optic nerve, which is mainly supplied by the pial plexus. PION is characterized by a normal optic disc and fundus findings in its initial phase. [17] A mild form of PION may be overlooked if patients suffer only small visual field defects unless they undergo ophthalmologic examinations that include detailed visual field tests.

IOP was significantly increased during RALP in this study as previously reported. [3,4,9] According to previous studies, visual field sensitivity significantly but reversibly decreases immediately after artificial elevation of IOP in eyes with and without glaucoma. [18] Amplitude of pattern electroretinogram was significantly decreased even in young normal eyes after slight and short time head-down position [19] and such changes were greater in the ocular hypertension eyes than in normal eyes with increased IOP. [20] These studies suggest temporary increased IOP and thereby impaired perfusion can affect visual function even in normal eyes, and glaucomatous eyes may be more vulnerable. IOP and perfusion are probably both involved in the impairment of visual function, and those mechanisms are hard to be separated.

The pathogenesis of ION after systemic surgery may be associated with severe arterial hypotension under general anesthesia or massive blood loss, hemodilution by overinfusion of intravenous fluid, or increased orbital pressure. [17] RALP is a minimally invasive procedure, but it requires a steep Trendelenburg position and IOP is substantially increased during the procedure, followed by a decrease in the ocular perfusion pressure. Although there are case reports of severe visual function loss after RALP, no prospective studies have been performed to examine the visual field in detail before and after RALP, and thus the clinical features and frequency of visual field defects after RALP are not clear. We believe this study is the first to prospectively evaluate the effect of RALP on visual field, as well as IOP, RNFL thickness, and optic disc morphology.

In this study, 25 subjects underwent RALP and 7 eyes of 7 subjects (28%; 95% confidence interval of 12–49%) showed significant visual field defects one week after surgery. None of the subjects were aware of the visual field loss, and none complained of any ocular symptoms, including visual acuity loss or anorthopia. The visual field returned to normal within 3 months after the surgery in all cases. Normal optic disc, macula, and clear optic media were confirmed by detailed ocular examinations in each eye. There were no abnormal findings in RNFL thickness or optic disc morphology measured using OCT or detailed funduscopy before and after RALP, and it is thus unlikely that those transient visual field defects were due to retinal pathologic changes, including macular edema or glaucoma.

Among the several risk factors of ION after surgical procedures, [17] amount of blood loss, infusion volume, and operation time did not differ significantly between subjects with and without visual field defects. Mean oxygen saturation were 99.1±0.8% and 99.2±0.7% and mean ET CO_2_ were 35.2±2.2 and 35.9±2.2 mmHg during the surgery in the subjects with and without postoperative visual field defects, respectively, and not significantly different between the two groups. The difference in blood loss was not significant between them even after standardization by body weight of each subject (P = 0.34). The blood loss remained in relatively small amount (mean of 267 ml and maximum of 695 ml) in the subjects of the current study, and it may still be possible that massive blood loss cause visual impairment. Correlations of the possible risk factors with the visual field were also analyzed in the both eyes of the all subjects using a linear mixed-effects model. Dependent variables were differences in MD or PSD between preoperative and initial postoperative visual field tests, and age, BMI, amount of blood loss, or time-integrated OPP during the surgery did not show any significant correlations. There was a trend for greater deterioration in postoperative MD with older age, however, this did not reach statistical significance (p = 0.063).

Recently, it has been reported that propofol has less influence on IOP than sevoflurane during RALP. [21] In the subjects of this study, desflurane was used in most of the cases in expectation of its rapid recovery especially in in elderly patients [22] and less postoperative cognitive dysfunction compared with propofol. [23] In the two propofol-treated cases, IOP increased up to 32 and 33 mmHg and mean IOP was not significantly different from those of desflurane-treated cases during the surgery and the postoperative visual field defect was found in one of them.

**Fig 3:**
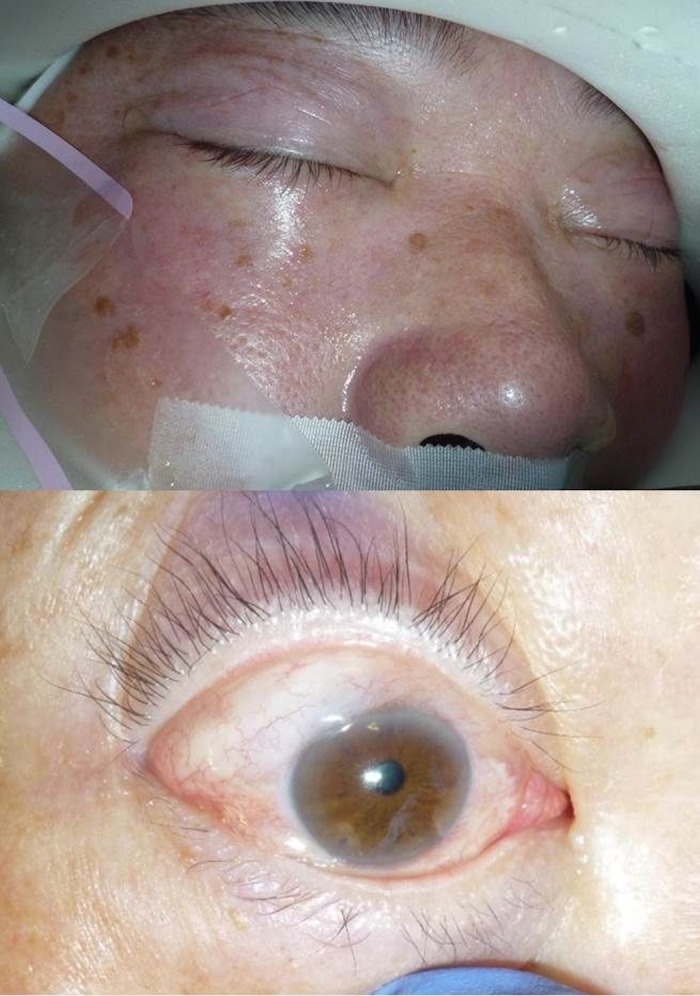
Typical appearance of the periocular swelling and chemosis. Moderate chemosis, lid swelling, and subcutaneous bleeding were observed dominantly in the superior part or the periocular tissues. (10 min after release of the head-down position).

Slight or moderate periocular swelling and chemosis were observed in all cases at the end of RALP (typical appearance shown in Fig 3), but these were completely absorbed within 0–2 days. Background risk factors for developing ION include diabetic mellitus, [24] smaller optic disc cupping, and smaller cup/disc ratio. [25,26] In this study, two patients had diabetic mellitus but neither developed postoperative visual field defects. Twelve patients had hypertension and four of them developed postoperative visual field defects. Mean blood pressure and OPP during the surgery was not significantly different between the subjects with and without hypertension. Average or sectored RNFL thickness or disc parameters, including disc size, cup/disc ratio, and cup volume were not significantly different between those with and without postoperative visual field defects in the preoperative examinations, and were not significantly altered after RALP. The subjects of this study are confined to a small number, and further study with larger sample size may reveal the significant risk factors of developing postoperative visual field defects.

Among the seven eyes with postoperative visual field defects, five had visual field defects in the lower hemifield and two had more centered visual field defects in both upper and lower hemifields (Fig 1). Visual field defects in the inferior field are most common in cases with anterior ION [27,28] and central scotomas are most common in cases with PION. [17] Hence, the visual field defects observed in the present study seemed to be consistent with AION or PION. The visual field spontaneously returned to normal in all 7 eyes, which is not consistent with ordinary ION cases after surgery. [17] Actually, most of the reported ION cases were initially diagnosed based on the sudden development of symptoms of manifest impairment in visual acuity or visual field, and visual field measured using kinetic Goldmann perimeter. [17] In contrast, none of the subjects in the present study had a visual acuity loss and their scotomas were only detected by scheduled visual field examinations using a static perimeter. A previous study using a static perimeter (Octopus Program 32) reported that a relatively large proportion (31.6%) of patients with NAION shows improvement in their visual field sensitivity, [29] indicating that NAION is at least partly reversible in the early phase.

In summary, we conducted a prospective study in 25 patients who underwent RALP. IOP significantly increased during the surgery and significant, but symptomless, visual field defects were detected by static perimetry in 7 eyes of 7 subjects 1 week later. There were no abnormal findings in the fundus, RNFL thickness, or optic disc morphology analyzed using OCT, and the impaired visual field recovered to normal 3 months after the surgery, suggesting ION or other ischemic factors as a possible mechanism. Operation time, blood loss, intraocular pressure during RALP, or optic disc parameters were not associated with the development of visual field defects. The results of the present study suggest that transient but significant visual field defects are a possible complication after even uneventful RALP. Predictive risk factors were not clear, however, indication for RALP should be cautiously considered in patients with risks of optic nerve perfusion, such as glaucoma or diabetic mellitus, and detailed ocular examinations are suggested before and after the surgery.
